# Impulsivity and compulsivity are differentially associated with automaticity and routine on the Creature of Habit Scale

**DOI:** 10.1016/j.paid.2019.07.003

**Published:** 2019-11-01

**Authors:** Karen D. Ersche, Laetitia H.E. Ward, Tsen-Vei Lim, Roderick J. Lumsden, Steven J. Sawiak, Trevor W. Robbins, Jan Stochl

**Affiliations:** a Departments of Psychiatry, Psychology, Clinical Neurosciences, University of Cambridge, Cambridge, UK; b Department of Kinanthropology, Charles University, Prague, Czech Republic

**Keywords:** Frequency, Personality trait, Questionnaire, Goal-directed, Mediation, Suppression

## Abstract

Habits may develop when meaningful action patterns are frequently repeated in a stable environment. We measured the differing tendencies of people to form habits in a population sample of n = 533 using the Creature of Habit Scale (COHS). We confirmed the high reliability of the two latent factors measured by the COHS, automaticity and routines. Whilst *automatic* behaviours are triggered by context and do not serve a particular purpose or goal, *routines* often have purpose, and because they have been performed so often in a given context, they become automatic only after their action sequence has been activated. We found that both types of habitual behaviours are influenced by the frequency of their occurrence and they are differentially influenced by personality traits. Compulsive personality is associated with an increase in both aspects of habitual tendency, whereas impulsivity is linked with increased automaticity, but reduced routine behaviours. Our findings provide further evidence that the COHS is a useful tool for understanding habitual tendencies in the general population and may inform the development of therapeutic strategies that capitalise on functional habits and help to treat dysfunctional ones.

## 1 Introduction

Habits are repetitive, meaningful actions in our daily lives, which often go unnoticed due to their automatic nature (Robbins & Costa, 2017). The scientific interest in habits has increased in recent years, not least because habits can make behaviour either highly efficient or severely dysfunctional and distressing. Understanding the factors that influence habit formation for the better and for the worse may thus have implications both for enhancing performance when habits are to our benefit, and developing treatments for habits that have become maladaptive. People also differ in their readiness to form and engage in habits in their daily lives, which we refer to as habitual tendencies. There is widespread agreement that habits form over time when behaviour is repeated regularly in the same context. Meanwhile, control over this behaviour gradually shifts from being guided by intentions to being automatically triggered by cues in the environment (see Wood & Runger, 2016). An example would be a person's tendency to take their shoes off automatically by the front door when returning from work. Importantly, habits are not restricted to single actions but may also involve sequences of actions, as exemplified by a night time routine to always prepare one's clothes for the next day before going to bed. Although this deferral of control to environmental stimuli can make habits highly functional by providing structure, reducing uncertainty and freeing up cognitive resources, it also makes behaviour less flexible, since much more effort is required to break or adjust a habit (Wood & Runger, 2016). In people with problems of regulatory control, habits may run the risk of spiralling out of control. A prime example is obsessive-compulsive disorder (OCD), where rather mundane habitual behaviour patterns such as washing one's hands or locking the front door when leaving the house become problematic for the afflicted individual. Patients with OCD find themselves unable to stop performing certain routines, even when they become dysfunctional and negatively affect their lives (American Psychiatric Association, 2013). Another example is drug addiction, where individuals lose control over the initiation, amount and duration of their habitual drug use, and pursue their drug-taking routines even in the face of extremely adverse consequences (American Psychiatric Association, 2013).

Experimental research in both animals and humans has been trying to elucidate the mechanisms underlying habit formation, and to identify factors that may precipitate the development of habits. External factors such as exposure to stimulant drugs (Corbit, Chieng, & Balleine, 2014; Ersche et al., 2016; Gourley, Olevska, Gordon, & Taylor, 2013; Nelson & Killcross, 2006), excessive training (Colwill & Triola, 2002; Holland, 2004; Thrailkill, Trask, Vidal, Alcala, & Bouton, 2018) and stress (Dias-Ferreira et al., 2009; Schwabe & Wolf, 2009) have all been shown to facilitate the formation of habits. Thus, behaviour that is regularly performed in a state of either acute or chronic stress is more prone to become habitual and controlled by environmental stimuli (Dias-Ferreira et al., 2009). For example, individuals with a history of stressful life events who also use drugs recreationally, are highly likely to develop a drug-taking habit when they use drugs regularly in the same context (e.g. always in a club on a Friday night). Even if these individuals do not have the intention to use drugs, going to a club on a Friday night increases the likelihood that they will be using drugs that night. As habitual behaviour is triggered by the context, it is the environment that prompts their drug use, overriding their intentions. Habitual drug use does not, however, equate to addiction, but internal factors such as impulsive personality traits have been shown to precipitate – at least in animal models of addiction – the transition of initially adaptive habits into maladaptive ones (Belin, Mar, Dalley, Robbins, & Everitt, 2008). Impulsive individuals are thus at risk of their drug-taking habits spiralling out of control and becoming compulsive. This means that they may continue using drugs even if this poses an acute threat to their health, professional or social life, such as by risking myocardial infarction, job loss, or relationship breakdown. The exact mechanism underlying the transition from functional to dysfunctional habits is, however, still unclear, but as there is a great need for effective treatments for individuals who have lost control over their habits, as well as for strategies to promote habit formation in individuals affected by cognitive decline or dementia who struggle to manage their daily lives, the interest in understanding habitual behaviours will continue to increase.

Healthy individuals in the general population who describe themselves as ‘creatures of habit’ are therefore of particular interest for research as they show an increased propensity to develop habits without them becoming pathological. We recently developed the Creature of Habit Scale (COHS, Ersche, Lim, Ward, Robbins, & Stochl, 2017) to measure individual variation in habitual tendencies in the general population. We confirmed modulatory effects of stimulant drug use and life adversity on habitual tendencies, as assessed by the COHS. We also identified positive relationships between compulsivity and habitual tendencies, but at the time we did not examine relationships with impulsivity (Ersche et al., 2017). Impulsivity and compulsivity are two personality traits associated with a lack of control over behaviour, which might explain why in some people automatic habits risk spiralling out of control. Whilst *impulsivity* reflects a failure to inhibit the initiation of behaviour, *compulsivity* characterizes a failure to stop an ongoing behaviour that is becoming inappropriate to the situation (see Robbins, Gillan, Smith, de Wit, & Ersche, 2012). Although both constructs reflect distinctly different deficiencies in the regulatory process, they may occur together.

We also did not investigate at the time the influence of participants' prior experience with each situation referred to in the questionnaire items. This is a fair concern given that habits develop gradually through associative learning and repetition (Wood & Neal, 2007), suggesting that the more often action patterns are performed in a given context, the greater the likelihood of the context triggering the actions automatically irrespective of the goal (Verplanken & Orbell, 2003). In the aforementioned example of drug-taking, it would be important to know whether it really matters how often people take drugs before their drug use becomes habitual, or whether predisposing personality factors that precipitate the development of habits are more important. In experimental settings, overtraining of an instrumental action is commonly used to induce stimulus-response habits (Adams & Dickinson, 1981; de Wit & Dickinson, 2009; Tricomi, Balleine, & O'Doherty, 2009). However, whilst in animal models the extent of training seems to be directly related to the predominance of the stimulus-response habit (Dickinson, 1985), such a relationship does not seem to hold for humans, according to the findings of experimental work (for review de Wit et al., 2018). It is therefore conceivable that the strength of human habits is not proportional to the extent of prior practice.

The aim of the present study was therefore to assess the extent to which the self-reported frequencies with which habitual actions have been performed accounts for habitual tendencies, as assessed by the COHS. We also aimed to investigate the effects of impulsivity and compulsivity on habitual tendencies in daily life. As the COHS is a relatively new measure, we used the opportunity to re-evaluate its psychometric properties in this new sample.

## 2 Methods

### 2.1 Sample

We recruited study participants through the online platform Amazon MTurk to examine variations in regular behaviours, since this platform has been regarded as suitable for obtaining data from the general population (Mortensen & Hughes, 2018). Participation requirements were a minimum age of 18 years and current residency within the United States of America. There were no restrictions with respect to gender, ethnicity or employment status. We also collected background information, including ethnicity, native language, education level, and employment status. A total of 565 participants completed the study, but data of 32 participants (6%) had to be excluded post hoc due to invalid responses or inattentive responding [assessed through recommendations by Meade & Craig, 2012]. Excluded individuals did not differ from the remaining sample on any demographic variable. The final sample included 533 participants with a mean age of 36.8 years [±10.8 standard deviation (SD), age range 18–71 years]. The sample was almost evenly split between male (50.8%) and female (49.2%) participants, of whom 81% identified as Caucasian, 9% as African American, 4% as Asian, 4% as Hispanic, and 2% as Multiracial. The overwhelming majority of participants were native English speakers (99%), who were at the time of the study in full-time employment (70%) [15% in part-time employment, 13% not in paid work, and 2% studying].

### 2.2 Procedures

The study was approved by the School of Biological Sciences Research Ethics Committee (PRE.2015.124; PI: KD Ersche). Participants received $3.00 for the completion of the study, which included the assessment of functional habits using the COHS questionnaire (Ersche et al., 2017), which includes 27 statements to which participants indicate their level of agreement on a 5-point Likert scale, ranging from strongly disagree (1) to strongly agree (5). Once participants completed the COHS, they were asked to indicate how often they engage in the behaviour described by each item by selecting one of the following response options: never, once a month, twice a month, three times a month, once a week, twice a week, three times a week, four times a week, five times a week, six times a week, once a day, twice a day, three times a day, four times a day, five times a day, or more than five times a day. These responses were then coded respectively from 0 (never) to 15 (five times a day). This fine-grained scale was deliberately selected to capture the wide spread of individual lifestyles as it had been previously used to quantify habitual behaviours (Verplanken & Orbell, 2003).

In order to determine participants' levels of trait impulsivity, we administered the *Barratt Impulsiveness Scale* (BIS-11, Patton, Stanford, & Barratt, 1995). The BIS-11 is a widely-used 30-item questionnaire that measures impulsive personality traits in three dimensions: attention (inattention and cognitive instability), motor behaviour (spontaneous actions), and non-planning (lack of forethought). As a personality trait, impulsivity covers the spectrum from normal to maladaptive behaviour, and it can be assessed using the same tool in both healthy people and patients. The following cut-off scores have been suggested to differentiate variation in trait-impulsivity: BIS-11 total scores between 52 and 71 are indicative of the normal range of impulsivity (n = 296, 56%), scores below 52 reflect individuals who are extremely over-controlled (n = 176, 33%), and scores above 72 signify highly impulsive individuals (n = 61, 11%) (see Stanford et al., 2009).

For the assessment of compulsive tendencies, we administered the *Obsessive-Compulsive Inventory–Revised* (OCI-R, Foa et al., 2002), which requires participants to rate 18 common obsessive-compulsive symptoms in terms of the degree to which they have been bothered or distressed by them in the past month on a 5-point scale, ranging from not at all (0) to extremely (4). Whilst subclinical levels of obsessive-compulsive symptoms are common in the general population (Sher, Martin, Raskin, & Perrigo, 1991), an OCI-R score of 21 or more suggests obsessive-compulsive symptoms of clinical severity (Foa et al., 2002). In the present sample, the vast majority of participants (n = 425, 80%) scored within the normal range, whilst the scores of 20% of participants (n = 108) pointed toward particularly high levels of compulsivity.

### 2.3 Factor structure of the Creature of Habit Scale (COHS)

We used confirmatory factor analysis to verify that the COHS structure consists of two latent factors (automaticity and routine) as reported in our previous study (Ersche et al., 2017). Means and variances adjusted Weighted Least Squares (WLSMV) were used as estimators in all presented models. Fit was evaluated using traditional indices such as Root Mean Square Error of Approximation (RMSEA), Comparative Fit Index (CFI) and Tucker-Lewis Index (TLI). Reliability of subscales was assessed by McDonald's omega (McDonald, 1999), and for reasons of convention, we also computed Cronbach's alpha (Cronbach, 1951).

**Fig. 1:**
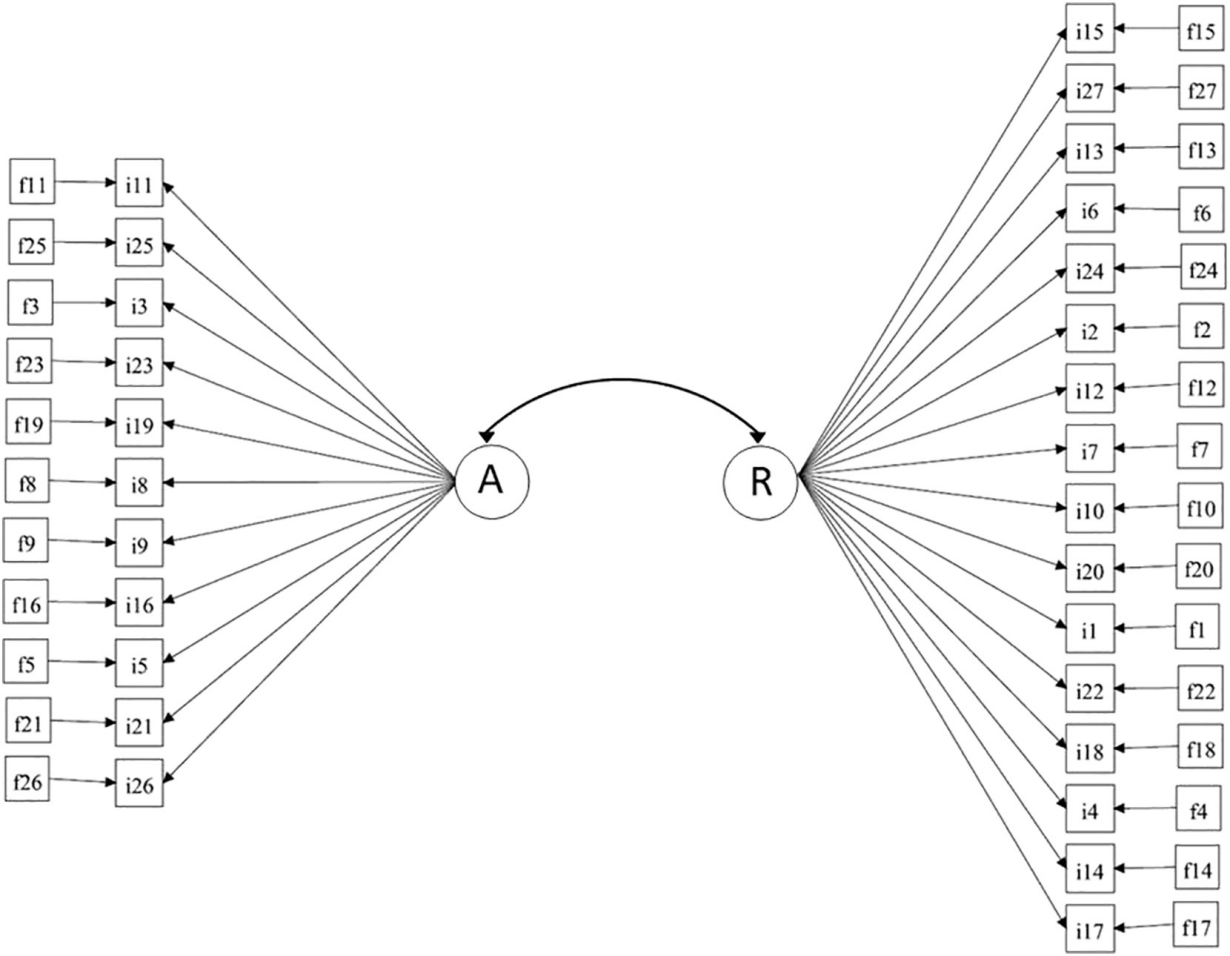
Conceptual path diagram of the COHS model, consisting of the two factors automaticity (A) and routine (R) adjusted for frequency (f) for each item of the COHS (i).

We extended our factor analytic model by regressing each COHS item onto the corresponding frequency item. The conceptual path diagram of this model is shown in Fig. 1. The rationale for this approach was two-fold: firstly, it allowed us to quantify the influence of frequency on COHS items (by using difference in R-squares of COHS items between frequency-adjusted and non-adjusted factor analytic models). Secondly, we evaluated whether the constructs of automaticity and routine hold whilst taking into account the frequency with which participants previously engaged in these behaviours.

### 2.4 Relationship between impulsivity and compulsivity

We further extended the CFA model by including self-reported impulsivity (BIS-11) and compulsivity (OCI-R) levels as predictors of the latent factors for automaticity and routine. We used participants' individual responses on the COHS without any adjustments for frequency. Our aim was to investigate how levels of impulsivity and compulsivity relate to participants' responses with respect to automaticity and routine. We estimated the models using the statistical software Mplus, version 8 (Muthén & Muthén, 2018) and standardized the results.

**Fig. 2:**
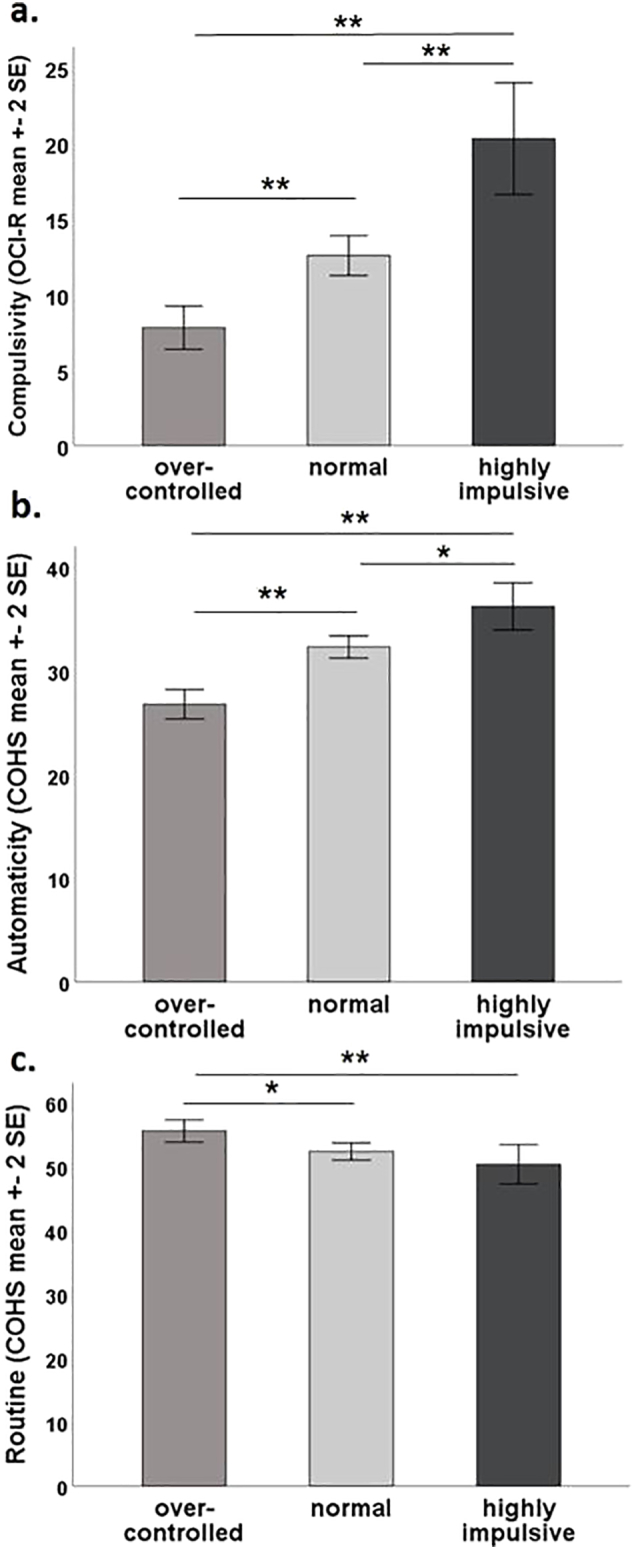
Levels of compulsivity, automaticity, and routine behaviours across individuals with varying levels of impulsivity (as subdivided according to the BIS-11 cut off score for over-controlled, normal, and highly impulsive levels). A Jonckheere-Terpstra test for ordered alternatives showed statistically significant trends for levels of low, normal and high trait-impulsivity for both higher mean OCI-R scores (TJT = 51.93, z = 7.24, p < 0.001) and high mean COHS automaticity scores (TJT = 54.20, z = 7.53, p < 0.001). For routine behaviours (COHS), a significant trend in the opposite direction was found (TJT = 33.97, z = −3.455, p < 0.001).

Solely for descriptive purposes, we divided the sample into three subgroups reflecting the three categories of impulsivity, as measured by the BIS-11. For illustrative purposes only, we compared these subgroups with respect to compulsivity (OCI-R) and habitual tendencies (COHS automaticity and routine) using the Kruskal-Wallis and the Jonckheere's trend tests to identify differences and trends respectively (Fig. 2).

## 3 Results

### 3.1 Factor structure of the Creature of Habit Scale (COHS)

**Fig. 3:**
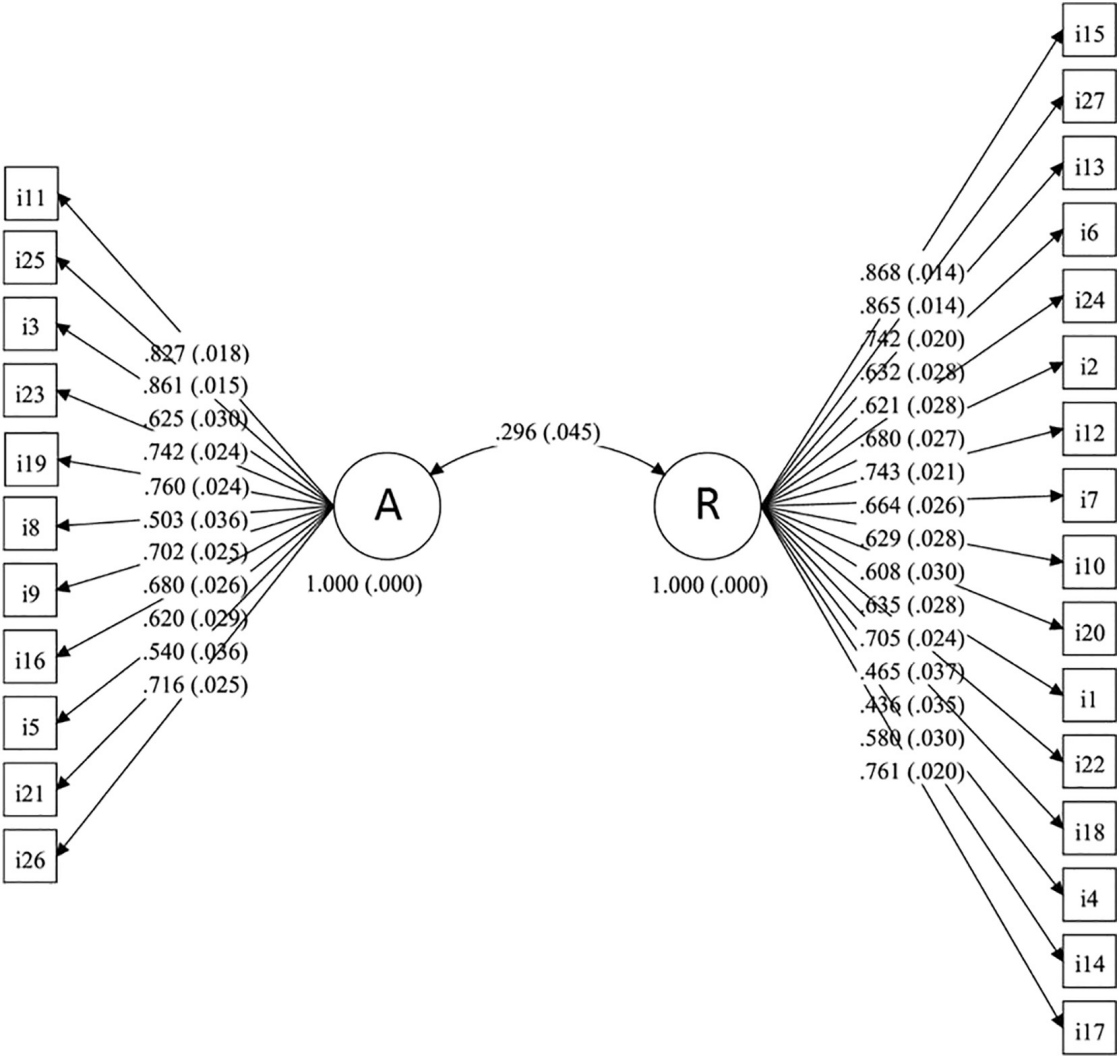
Path diagram of COHS model with standardized loadings (standard errors in brackets). [Note: A stands for COHS automaticity and R for COHS routine; i stands for the individual COHS items and f for the frequency rating of each of these items.]

The two-factor structure of the COHS fitted the data well (Chi-square(df) = 1169(323), p ≤0.001, RMSEA = 0.070, 90%CI for RMSEA = (0.066, 0.075), CFI = 0.977, TLI = 0.975), supporting the notion of automaticity and routine being two subscales of COHS. Both factors were also moderately correlated with each other (r = 0.296, p < 0.001). The loading of both factors was high, indicating high factorial validity of items. Likewise, the reliability of the coefficients for both factors was high as well, i.e. COHS routine (Cronbach's alpha: 0.90; McDonald's omega: 0.94) and COHS automaticity (Cronbach's alpha: 0.87; McDonald's omega: 0.92), providing support for satisfactory measurement precision of both subscales (Fig. 3).

### 3.2 The influence of frequency on the COHS scores

The inclusion of the frequency ratings in the model also fitted the data well (RMSEA = 0.032, CFI = 0.93, TLI = 0.93). In this model, factor loadings were adjusted for the influence of frequency ratings. Model estimates are shown in Table 1. In brief, the loadings were smaller compared to the model without frequency, but remained high enough to support the existence of two underlying factors. This suggests that habits, as conceptualised in the COHS, cannot be fully explained by how frequently the corresponding activities have previously been carried out.

**Table 1:** Standardized estimates (SE) of the model both with and without frequency ratings of COHS items.

	COHS item	Factor loading	SE	p-Value	Frequency rating		
					Regression	SE	p-Value
					Estimate		
Routine	**Item 15**: I tend to like routine.	0.65	0.03	<0.001	0.37	0.06	<0.001
	**Item 27**: I find comfort in regularity.	0.63	0.03	<0.001	0.47	0.05	<0.001
	**Item 13**: I rely on what is tried and tested rather than exploring something new.	0.69	0.02	<0.001	0.09	0.06	0.151
	**Item 6**: I quite happily work within my comfort zone rather than challenging myself, if I don't have to.	0.52	0.03	<0.001	0.34	0.05	<0.001
	**Item 24**: I tend to stick with the version of the software package that I am familiar with for as long as I can.	0.53	0.03	<0.001	0.22	0.06	<0.001
	**Item 2:** I generally cook with the same spices/flavourings.	0.53	0.03	<0.001	0.55	0.04	<0.001
	**Item 12**: I normally buy the same foods from the same grocery store.	0.62	0.03	<0.001	0.28	0.06	<0.001
	**Item 7**: I tend to do things in the same order every morning (e.g. get up, go to the toilet, have a coffee…).	0.49	0.04	<0.001	0.39	0.06	<0.001
	**Item 10**: I always try to get the same seat in places such as on the bus, in the cinema, or in church.	0.51	0.03	<0.001	0.42	0.05	<0.001
	**Item 20**: I usually sit at the same place at the dinner table.	0.43	0.04	<0.001	0.53	0.04	<0.001
	**Item 1:** I like to park my car or bike always in the same place.	0.45	0.04	<0.001	0.47	0.05	<0.001
	**Item 22**: I always follow a certain order when preparing a meal.	0.59	0.03	<0.001	0.40	0.05	<0.001
	**Item 18**: I am one of those people who get really annoyed by last minute cancellations.	0.33	0.04	<0.001	0.36	0.05	<0.001
	**Item 4**: I tend to go to bed at roughly the same time every night.	0.33	0.03	<0.001	0.65	0.03	<0.001
	**Item 14**: I generally eat the same things for breakfast every day.	0.44	0.03	<0.001	0.63	0.03	<0.001
	**Item 17**: In a restaurant, I tend to order dishes that I am familiar with.	0.70	0.03	<0.001	0.22	0.06	<0.001
Automaticity	**Item 11**: I often find myself finishing off a packet of biscuits just because it is lying there.	0.71	0.03	<0.001	0.21	0.06	<0.001
	**Item 25**: I often find myself opening up the cabinet to take a snack.	0.74	0.03	<0.001	0.29	0.06	<0.001
	**Item 3**: When walking past a plate of sweets or biscuits, I can't resist taking one.	0.40	0.04	<0.001	0.48	0.04	<0.001
	**Item 23**: Television makes me particularly prone to uncontrolled eating	0.61	0.03	<0.001	0.19	0.06	0.001
	**Item 19**: I often find myself eating without being aware of it.	0.61	0.03	<0.001	0.35	0.06	<0.001
	**Item 8**: Eating crisps or biscuits straight out of the packet is typical of me.	0.34	0.04	<0.001	0.51	0.04	<0.001
	**Item 9**: Whenever I go into the kitchen, I typically look in the fridge.	0.45	0.03	<0.001	0.59	0.04	<0.001
	**Item 16**: I usually treat myself to a snack at the end of the workday	0.55	0.03	<0.001	0.20	0.05	<0.001
	**Item 5**: I often take a snack while on the go (e.g. when driving, walking down the street, or surfing the web).	0.43	0.04	<0.001	0.50	0.04	<0.001
	**Item 21**: I often find myself running on ‘autopilot’, and then wonder why I ended up in a particular place or doing something that I did not intend to do.	0.50	0.04	<0.001	0.46	0.04	<0.001
	**Item 26**: I am prone to eating more when I feel stressed.	0.40	0.04	<0.001	0.62	0.04	<0.001

Standardized regression coefficients (adjusted for relationships between items and the corresponding factors) between the COHS items and the corresponding frequency ratings ranged from 0.09 to 0.65. Except item 13 (*I rely on what is tried and tested rather than exploring something new*.), all COHS items were statistically significant, suggesting that participants' responses can be partially, but not fully, explained by the self-reported frequency with which the behaviour in question has been repeated. The average difference between R-squares for frequency-adjusted and non-adjusted factor models was 0.15 for automaticity items and 0.11 for routine items, suggesting that frequency explains slightly more variance for automaticity than for routine.

### 3.3 The effects of deficient regulatory control on habitual tendencies

**Fig. 4:**
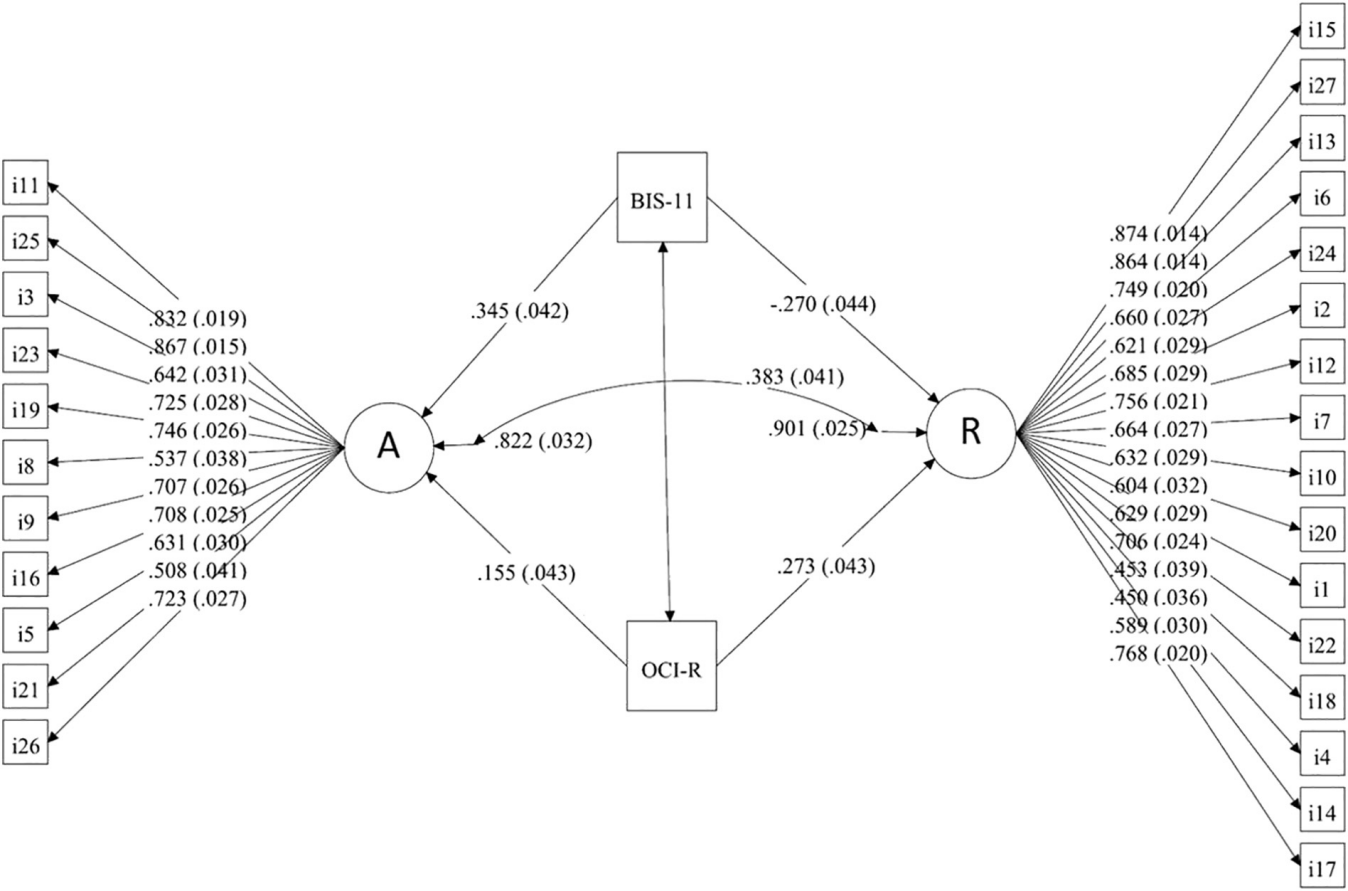
Model of the relationship between the two COHS factors *automaticity* (A) and *routine* (R) and personality traits of impulsivity (BIS-11) and compulsivity (OCI-R).

Fig. 4 depicts the model we used to evaluate the effects of insufficient regulatory control, as reflected by self-reported measures of impulsivity and compulsivity. All factor loadings, regressions and correlations included in this model were statistically significant (all p-values <0.001). Automaticity was positively related to both impulsivity and compulsivity, suggesting that participants with higher levels of impulsivity and/or compulsivity are more likely to report increased tendencies for automaticity. It is noteworthy that the effect of impulsivity (β = 0.345) on automaticity was larger than the effect of compulsivity (β = 0.155). For routine behaviours, we observed relationships of approximately the same magnitude but of different directions with respect to impulsivity and compulsivity (β_impulsivity_ = −0.270 versus β_compulsivity_ = 0.273). This may indicate that more compulsive individuals are more prone to routine behaviours. This effect is, however, attenuated in individuals who are also impulsive, as impulsivity prevents the occurrence of routines.

## 4 Discussion

Habitual responses are part of everyday life, but the propensity to form habits differs substantially across individuals. Here we provide evidence for the validity of the two-factor model underlying the COHS for assessing habitual tendencies in the general population. We further confirm that the COHS is consistent with the theoretical concept of habits, which explains the formation of habits through a process of context-dependent repetition (Robbins & Costa, 2017; Wood & Runger, 2016). Both scales of the COHS were significantly influenced by the frequency of past behaviour but, importantly, they were not fully explained by it. Our findings thus not only concur with prior experimental work in humans (de Wit et al., 2018), they also have important implications for the use of the COHS, making it a potentially useful psychometric instrument for investigating habitual tendencies in the general population.

Our data further suggest that habitual tendencies are related to personality traits, specifically those that characterise an individual's disposition in regulating behaviour. Whereas both impulsivity and compulsivity were positively related to automaticity, they had conflicting associations with the tendency to routine. Impulsivity was negatively associated with routine behaviours, perhaps because of impaired behavioural regulation with respect to timing. By contrast, compulsivity, perhaps unsurprisingly, was positively associated with both routine and automaticity.

### 4.1 Frequency of past behaviour and habitual tendencies

There is widespread agreement that habit formation is a process, rather than an event, which implies that the repetition of behaviour is to some degree necessary for it to become habitual. This notion is also reflected in our data suggesting that habits are dependent on repetition but are not fully explained by it. What may seem self-evident has, however, not always been seen in this way. A decade ago, habits were understood as a combination of both *frequency* (as defined by repetition) and *automaticity* (as defined by a lack of control and awareness) (Verplanken & Orbell, 2003). Whilst repetition was deemed critical in the early stages of habit formation, automaticity was thought to play a more prominent role in the final stages of habit formation by implicitly ensuring that behaviour will be repeated once the habitual response has been established (Gardner, 2012; Verplanken, 2006). Clearly, repetition of behaviour through practice is needed for habit formation, but well-practised behaviours are not necessarily habits. Our two-step approach of composing the COHS questionnaire, first to select items of sufficiently general nature, and subsequently to collect frequency information for each of them, has allowed us to control statistically for the effects of repetition. As this study shows, both COHS scales clearly survived the corrections for different frequencies between individuals, thereby further supporting the notion that habitual tendencies are more than merely the frequency of their occurrence. Therefore, as recommended by Ajzen (2002), we should not solely rely on behavioural frequencies as the defining criterion for habits, but rather focus on the qualitative characteristics of habits, such as the declining influence of cognitive factors and the increasing control of stimulus cues over behaviour.

#### 4.1.1 Personality and proneness to habit

As habit formation is characterised by a devolution of control from intentions to the contextual cues, individuals with problems in self-regulation run the risk of automatic habits getting out of control. Our data suggest that trait impulsivity promotes habitual behaviour by facilitating this regulatory imbalance between the goal-directed and the habit system. Impulsivity seems to selectively enhance automatic stimulus-driven, goal-independent actions whilst diminishing the occurrence of routine behaviours. Our findings seem to complement previous findings in regular smokers, whose levels of trait impulsivity predicted how likely they would pick up a cigarette if they were craving for one (Hogarth, 2011). Conscious cravings predicted smoking only in less impulsive smokers, whereas in highly impulsive individuals smoking was more habitual since it was decoupled from the conscious desire for a cigarette. This subtle difference between goal-independence, as reflected by the COHS automaticity scale, and some relatedness of routines to a goal, appears to be critical in defining the influence of trait impulsivity on habit formation. Given that impulsive actions are spontaneous, premature, lack forethought, and have previously been described as an ‘inability to control automatic reactions to stimuli’ (Kopetz, Woerner, & Briskin, 2018), the positive relationship with stimulus-driven behaviours captured by the COHS automaticity scale may thus seem intuitive. The negative influence of impulsivity on routine behaviours, however, might be less obvious. Clearly, routines such as going to church on Sunday morning or brushing your teeth before going to bed are not fully independent from a goal, but are meaningful familiar action patterns, which have been performed many times in the same context so that they become automatic once the action sequence has been activated by the cognitive representation of the goal (Aarts & Dijksterhuis, 2000). Consequently, the difficulty that impulsive individuals have in adjusting the optimal timing for an action (as they initiate actions prematurely) is opposed to adhering to regularity and developing routines. In fact, the implementation of family routines is explicitly recommended to parents of impulsive children as routines provide them with a stable context in which consequences become more predictable, encouraging them to act less impulsively (Lanza & Drabick, 2011).

Compulsivity, by contrast, was positively associated with both aspects of habits, routine behaviours and automaticity, albeit to a lesser degree than impulsivity. The positive relationship is not surprising given that routines, at least those routines that have an almost ritualistic nature, may run the risk of developing into compulsive behaviours, as exemplified in obsessive-compulsive disorder (Mell et al., 2005). Likewise, the unconscious, stimulus-driven nature of compulsions has often been described by patients with compulsive disorders (McCusker & Gettings, 1997). It is thus conceivable that compulsive personality traits may enhance habitual tendencies under conditions of insufficient inhibitory control.

### 4.2 Habit formation from a neuroscientific perspective

Our findings also agree well with the substantial work in both animals and humans that has been defining the neural circuit architecture underlying the neuroadaptive processes of habit formation (Balleine & Dickinson, 1998; Tricomi et al., 2009; Yin, Knowlton, & Balleine, 2006). There is now broad consensus that habitual responding develops through the progressive engagement of different striatal subsystems along a ventral to dorsal gradient (Burton, Nakamura, & Roesch, 2015). During the early stages of habit formation, behavioural control relies heavily on dopaminergic pathways modulating interactions of the associative striatum and the lateral and medial prefrontal cortices, which encode the value of expected outcomes (Haber & Knutson, 2009). As during this stage behaviour is mainly goal-directed and performed on purpose, actions are selected on the basis of their anticipated consequences. With prolonged practice, however, the regulation of these actions is reorganised as behaviour becomes increasingly automatic and less dependent on dopaminergic neurotransmission in the aforementioned regions (Ashby, Turner, & Horvitz, 2010). This change is hypothetically underpinned by devolution of behavioural control to the sensorimotor striatum (Ashby et al., 2010). During this transition, information about action sequences is clustered together to form units of behavioural repertoires (or ‘chunks’), which can be quickly and more efficiently implemented (Graybiel, 1998). In other words, the sensorimotor system acts on the signals it receives from the prefrontal cortex and does not further evaluate how appropriate they are. Consequently, behaviour is executed irrespective of its potential consequences. Experimental evidence further shows that the extent of training (or repetition) is directly linked to changes in dopamine signalling, which induces the transition of control from the associative system to the sensorimotor system (Choi, Balsam, & Horvitz, 2005). Thus, from a neuroscientific point of view, the repetition of behaviour is not equivalent to habit, but represents an essential ingredient for its development.

With respect to the influence of impulsive and compulsive traits on habit formation, there is also growing evidence of impulsivity being associated with deficits in goal-directed control (Gillan, Kosinski, Whelan, Phelps, & Daw, 2016; Hogarth, Chase, & Baess, 2012). Consequently, reduced prefrontal involvement during goal-directed choices may facilitate habitual responding (Deserno et al., 2015). The neural substrates of compulsivity have been associated with dysregulation of control functions implemented by frontostriatal and corticostriatal circuitries (van den Heuvel et al., 2016). Subclinical levels of compulsivity in healthy volunteers seem to be associated with a volume enlargement of the putamen (Kubota et al., 2016), a structure that is significantly enlarged in patients with compulsive disorders (Chamberlain et al., 2008; Ersche et al., 2011, Ersche et al., 2012; Pujol et al., 2004), suggesting a highly active sensorimotor system (Kwon et al., 2003) that facilitates habit formation.

### 4.3 Strengths, weaknesses and outlook for further research

Strengths of the study include the relatively large sample size, the re-evaluation of the COHS's psychometric properties (given that it is a relatively new tool), and our aim to address the theoretically important question about the influence of frequency on habit formation. The latter is particularly pressing for assessing habitual tendencies by questionnaire given the discrepancies between animal and human experimental work. Due to practical constraints of this online study, the occurrence of habitual behaviours and their frequencies could only be assessed by self-report and not verified objectively, which should be addressed by future studies. We used a 15-point frequency scale to allow for the variation in lifestyles in our sample. We acknowledge that for some COHS items, frequency data are more difficult to collect than for others. For example, items such as ‘I like routines’, may not spark an obvious answer but our pilot work suggests that participants understood this item in terms of the frequency with which they generally obtain gratification when engaging in their routines, which is consistent with the item's meaning. Our finding that frequency is important but not sufficient for the formation of habits is critical for the assessment of habits by questionnaire more generally, given that habits are dependent on people's lifestyles, which determine how frequently they engage in a behaviour. The COHS items were therefore carefully chosen, and as suggested by the present study, are not compromised by how frequently respondents report having previously engaged in the behaviour in question. Our findings are thus in keeping with both the theoretical concept of habits and the neuroscientific evidence of their development.

The COHS has not been designed to measure habit strength in terms of how persistently an individual pursues a particular habit, but rather to assess individual variation in readiness to engage in habitual behaviours in daily life. It should therefore not be mistaken with the Self-Reported Habit Index (Gardner, De Bruijn, & Lally, 2011; Verplanken & Orbell, 2003), which requires individuals to rate their experience with the regular behaviour in question. Further work is, however, warranted to investigate how proneness to habits expresses itself during habit learning in an experimental paradigm. It would be of particular interest to test whether high COHS scores are predictive of enhanced acquisition of stimulus-response habits and the (in)ability to adjust them according to changing circumstances. The present study does not necessarily indicate that the proneness to form functional habits is a risk marker for the transition to dysfunctional habits; however, we would welcome research in patients with dysfunctional habits to investigate whether the COHS might inform preventative or therapeutic strategies for these patients.

## 5 Conclusions

This present study examined the inter-relationships between three potentially related traits: impulsivity, compulsivity and the proneness to habits, defined according to two distinct criteria, automaticity (goal-independent) and routine (goal-related), and not dependent on their frequency of occurrence. Whereas both impulsivity and compulsivity are associated with increased automaticity of behaviour, they may thus exert opposite effects on routine habitual behaviour, impulsivity being negatively correlated. These findings may be relevant for identifying vulnerability to certain mental health disorders and for understanding how learning may become aberrant and produce psychopathology.

## Declaration of Competing Interest

All authors declare that they have no competing or potential conflicts of interest in relation to this work.
